# Investigating the protective effects of mindfulness-based attention training on mind wandering in applied settings

**DOI:** 10.3389/fpsyg.2023.1232598

**Published:** 2023-12-28

**Authors:** Malena M. Price, Anthony P. Zanesco, Ekaterina Denkova, Jordan Barry, Scott L. Rogers, Amishi P. Jha

**Affiliations:** ^1^Department of Psychology, University of Miami, Coral Gables, FL, United States; ^2^School of Law, University of Miami, Coral Gables, FL, United States

**Keywords:** off-task thoughts, cognition, sustained attention, workplace, mind wandering

## Abstract

**Introduction:**

Mind wandering, a phenomenon in which attention drifts away from the task-at-hand, is associated with deleterious effects on performance and well-being. As such, efforts to curb mind wandering are warranted. Recently, mindfulness training (MT) has been found to protect against mind wandering. Yet, many MT programs are at risk of falling off the *implementation cliff* due to challenges implementing these programs in applied settings. To mitigate against this, early-stage research in small convenience samples may be necessary to spur stakeholder engagement and collaboration. Herein, the effects of MT on mind wandering were examined via an internal meta-analysis of early-stage studies of a manualized, context-adaptable short-form MT program, referred to as Mindfulness-Based Attention Training (MBAT).

**Methods:**

Five longitudinal studies (*N* = 304) were conducted in a variety of organizational cohorts. Self-reported mind wandering and meta-awareness, as well as accuracy (*A’*) and response time variability (intra-individual coefficient of variation, ICV) during performance of the sustained attention to response task (SART) were assessed at baseline (T1) and 4 weeks later (T2) in MBAT and no-training participants.

**Results:**

Standardized mean change (SMC) from T1 to T2 significantly differed between MBAT and no-training groups for mind wandering (ΔSMC = −0.387, *p* < 0.001), meta-awareness (ΔSMC = −0.374, *p* < 0.001), and ICV (ΔSMC = −0.376, *p* = 0.043), suggesting potential protective effects in self-reported and performance-based metrics of mind wandering.

**Discussion:**

These results serve as preliminary proof-of-concept support for MBAT’s protective effects on mind wandering. Further, they suggest that MBAT is amenable to implementation across a variety of applied and organizational settings and warrants additional research employing larger sample sizes in randomized controlled designs.

## Introduction

Mind wandering is a pervasive phenomenon characterized by attentional instability wherein off-task thoughts occur during an ongoing task or activity. Herein, we focus on mind wandering during cognitive task performance. One laboratory-based cognitive task during which mind wandering has been readily assessed is the Sustained Attention to Response Task (SART; Robertson et al., 1997). During this task, self-reported mind wandering is assessed via embedded experience sampling probe questions that aim to capture mind wandering in the moment. Prior research has reliably linked greater subjective mind wandering with poorer task accuracy (e.g., Randall et al., 2014; Kane et al., 2016) and greater response time variability (Zanesco et al., 2020). Beyond laboratory settings, the occurrence of mind wandering has been associated with errors in everyday activities (Baldwin et al., 2017) and may be particularly consequential in applied workplace settings (Li, 2022). Given these detrimental effects, there has been growing interest in effective methods to promote greater attentional control over mind wandering.

One promising approach to reduce mind wandering is mindfulness training (MT; Feruglio et al., 2021; Turkelson and Mano, 2022). From a cognitive perspective, MT and related practices, are suggested to engage attentional skills involved in the regulation of mind wandering. For example, one category of MT practice—focused-attention practice—instructs practitioners to focus their attention on a specific target object (e.g., breath-related sensations), and to notice when their attention drifts away from this object to internal (i.e., thoughts) or external (e.g., a light flickering) distractions. When they catch themselves mind wandering, the practitioner is instructed to disengage from task-unrelated thought or other distractions and reorient attention back to the specific target object (see Lutz et al., 2015; Jha et al., 2019). With repeated engagement in MT and related practices, practitioners may strengthen processes (i.e., selective attention, monitoring, and disengagement) essential for noticing when the mind has wandered and redirecting attention back to the task-at-hand.

Prior studies involving intensive periods of MT, such as month-long retreats requiring full-time, daily mindfulness practice, report reduced mind wandering during performance on the SART (Witkin et al., 2022) and other tasks requiring sustained attention (e.g., Zanesco et al., 2016). In addition, mind wandering has been examined in studies involving mindfulness-based interventions (MBIs) such as 8-week programs frequently offered in healthcare settings (i.e., Mindfulness-Based Stress Reduction, MBSR; Kabat-Zinn, 1990; Giannandrea et al., 2019; and Mindfulness-Based Cognitive Therapy, MBCT; Teasdale et al., 2002; Greenberg et al., 2018), as well as shorter-form programs lasting 2 to 7 weeks (Mrazek et al., 2013; Levinson et al., 2014; Bennike et al., 2017). Across many of these studies, MT is found to be beneficial in reducing rates of mind wandering over the study interval.

Yet, in certain settings and cohorts, attentional control has been proposed to degrade over a few to several weeks, leading to greater mind wandering over the study interval. Recent studies in students tracked over the academic semester (e.g., Morrison et al., 2014), soldiers over pre-deployment training intervals (see Jha et al., 2020), and elite athletes engaged in intensive pre-season physical training (e.g., Rooks et al., 2017) report less mind wandering (e.g., Morrison et al., 2014; Rooks et al., 2017) over time in those receiving MT relative to comparison groups. Thus, in some settings, MT’s salutary effects may be to curb putative increases in mind wandering over time.

Protecting against mind wandering is of interest across a variety of applied and organizational settings in which its occurrence may be particularly hazardous for performance. While MT-related benefits have been observed with gold standard programs, there may be barriers for implementation related to program time demands, framing, and trainer competencies. As such, more research evaluating the efficacy of shorter, tailored MT programs in applied workplace settings is warranted (see Grégoire and Lachance, 2015).

A prominent framework that is often used to guide MT program development is the NIH stage model of behavioral intervention research (Onken et al., 2014). This model is typically utilized for clinical intervention development. The stages progress from basic research (i.e., Stage 0) to intervention development (i.e., Stage 1), efficacy (i.e., Stages 2–3), and effectiveness studies (i.e., Stage 4) to then culminate in real-world intervention dissemination (i.e., Stage 5). One challenge in intervention development is that intervention delivery interfaces with real-world settings during *later implementation stages*, which may disadvantage engagement and collaboration with stakeholders, and ultimately limit intervention adoption and uptake (see Loucks et al., 2021). Interventions that suffer from poor translation from research into real-world settings are described as falling off the “implementation cliff.” They suffer from low intervention uptake, as well as lower than predicted benefits as the intervention moves from research-controlled and monitored settings to real-world, community settings (Dimidjian and Segal, 2015; Griffith et al., 2021).

Herein, we describe a program of research investigating the effects of a specific MT intervention, Mindfulness-Based Attention Training (MBAT), on attentional performance and mind wandering. The overarching aim was to advantage MT implementation by interfacing with real-world, organizational cohorts in applied settings *earlier* in the stage progression of intervention development. While such an approach allows for greater initial engagement and collaboration with stakeholders to ensure contextual adaptation, there are acknowledged methodological tradeoffs with this emphasis. Sample sizes tend to be smaller in applied studies, and participants may be limited to convenience samples. In addition, experimental controls may be limited.

### Mindfulness-based attention training

MBAT has benefitted from prior Stage 0 and Stage 1 research. Stage 0 aims to identify the *targets* for intervention development. From a clinical perspective, the intervention target could be a specific symptom in a patient population (e.g., ruminative thinking in depressed patients). From a cognitive perspective, the target could be specific cognitive vulnerabilities that may have deleterious consequences in specific applied settings (e.g., mind wandering in task contexts or settings that require sustained focus for optimal performance). Stage 1 involves creating and adapting the intervention with these targets in mind, and then conducting preliminary testing of the intervention. In line with this approach, short-form MT programs were developed by our research team to examine their impact on mind wandering and task performance in various cohorts (Morrison et al., 2014; Rooks et al., 2017; Denkova et al., 2018). Informed by these and other prior studies of MT (Jha et al., 2010, 2017, 2022), MBAT was developed as a short-form, 4-week MT program that can be readily adapted for implementation in various applied and organizational settings.

Initial studies of MBAT have been conducted in military cohorts (Zanesco et al., 2019; Jha et al., 2020, 2022; Nassif et al., 2023). In addition, several studies of MBAT have been conducted in applied settings in civilian cohorts to date: including in firefighters, military spouses or relationship partners, corporate employees, community leaders, and educators. Studies varied in cohort-specific contextualization, randomization, and trainer type (embedded context-familiar or research affiliated trainers). While these are largely early-stage exploratory studies, they have the advantage of being contextualized and conducted in real-world, applied settings. In the present study, we conducted an internal meta-analysis of this body of research to determine if MBAT benefits attentional performance by taming mind wandering during ongoing task performance. The results of this meta-analysis will help determine whether further research with larger samples and rigorous randomized controlled trials is warranted.

## Materials and methods

Five longitudinal studies conducted by our research group are included in this internal meta-analysis (see Goh et al., 2016, for a discussion of internal meta-analyses, and Vosgerau et al., 2019, for a critical perspective). Procedures for all five studies were approved by the Institutional Review Boards at the University of Miami, and all participants provided informed consent prior to enrollment. One study was registered on ClinicalTrials.gov (Study 2; NTC03308344).

### Mindfulness-based attention training

Mindfulness-Based Attention Training (MBAT) is a manualized and structured program designed to allow for contextual adaptation within various time-pressured, applied settings. Prior studies have investigated MBAT delivery in military cohorts (Zanesco et al., 2019; Jha et al., 2020, 2022; Nassif et al., 2023). As described herein, the program has more recently been adapted for delivery in a variety of civilian settings (e.g., Denkova et al., 2020, 2021, 2022).

The MBAT course consists of 4, 2-h sessions delivered over 4 consecutive weeks, with 1 session per week. Each session introduces one of four central themes that progress in the following sequence: concentration, body awareness, receptivity, and connection. These themes are coupled with their four corresponding mindfulness exercises (focused attention, body scan, open monitoring, and connection practices, respectively). In addition, participants are asked to complete formal mindfulness exercises that correspond with the weekly course material as part of daily out-of-class individual mindfulness practice. After the first week of training, participants are instructed to alternate between the first week’s mindfulness exercise (i.e., focused attention) and the corresponding week’s newly introduced mindfulness exercise (e.g., Week 2: body scan). This modular and thematic structure is designed to maximize scheduling flexibility for course meetings, while maintaining content flow in applied, organizational settings. Delivery details are described in Table 1.

**Table 1:** Overview of included study design and MBAT conditions.

Study	Community context	MBAT format	Study condition assignment
Study 1	Firefighters	One 2-h class per week over 4 weeks	Cluster randomized controlled trial
Study 2	Military spouses	One 2-h class per week over 4 weeks	Non-randomized controlled trial
Study 3	Employees at a large company	Two 1-h classes per week over 4 weeks	Non-randomized controlled trial
Study 4	Community leaders	Two 4-h classes, weekly office hours over 4 weeks	Non-randomized controlled trial
Study 5	Middle and high school teachers	One 2-h class per week over 4 weeks	Cluster randomized controlled trial

Beyond scheduling flexibility, MBAT is designed to enable context-specific adaptation of the program to ensure that the themes, examples, and trainer-led discussions are relevant for the professional and lifestyle demands and challenges that specific cohorts may face (see Jha et al., 2020 and Denkova et al., 2021 for detailed descriptions of MBAT contextualized for soldiers and military spouses, respectively). While maintaining MBAT’s core mindfulness themes and practices, program materials are customized to incorporate context-relevant vernacular and examples. These adaptations are made in collaboration with community members, guided by the principles of community-based participatory research (Wallerstein and Duran, 2006). In addition, context-customization is achieved in the interactive program elements via MBAT’s train-the-trainer (TTT) dissemination model. Specifically, after participating in an MBAT teaching practicum, context-familiar trainers guide sessions to ensure that interactive discussions, participant questions, and guidance on how to best apply the MBAT themes to their lifestyle or situational challenges, benefit from trainers’ own embodied context familiarity (see Jha et al., 2020 for more details on the MBAT trainer practicum).

### Studies summary

Three of the five studies included herein have been published previously, and details regarding study designs can be found in their respective publications. The other two studies are unpublished. Study design characteristics are described below and summarized in Table 1.

#### Study 1

One hundred and twenty-one firefighters in South Florida were assigned by their work schedule/shift to receive MBAT (MBAT group: *n* = 42; *M* age = 43.61, SD = 8.23 years; 7 females), relaxation training (RT group; *n* = 31; *M* age = 45.38, SD = 6.80 years; 6 females), or to a no-training control (NTC group; *n* = 48; *M* age = 43.12; SD = 8.30 years; 10 females). In this study, while program materials were adapted for firefighters via collaboration with a community member, MBAT was delivered in person by a research-affiliated trainer. Both MBAT and RT participants were assigned 10–15 min of formal MBAT practices to be completed on a daily basis outside of class (see Denkova et al., 2020 for more details). This study used a cluster-randomized controlled design, in that assignment to MBAT, RT, or NTC was done according to the work shifts of firefighters per the requirements set by the Fire Department so that training and testing can be incorporated into participants’ workday shift schedules.

#### Study 2

In the Fall of 2018, 48 spouses or partners of military services members were assigned to receive mindfulness training (MBAT group; *n* = 48; *M* age = 37.60, SD = 6.61 years, 2 males), and in the Summer of 2019, 58 military spouses were assigned to a no-training control group (NTC group; *n* = 58; *M* age = 30.96, SD = 8.38 years; all females). The MBAT program was delivered in person by a context-familiar, non-research affiliated trainer. During the 4-week training interval, MBAT participants were assigned 10–15 min of formal, out-of-class MBAT practices. This study used a non-randomized design with the primary goal to examine the feasibility of MBAT delivery by peers who previously received a teaching practicum (see Denkova et al., 2021 for more details).

#### Study 3

Ninety-five employees from a large company in South Florida participated in this study. Of the ninety-five, fifty employees volunteered to participate in MBAT at work (MBAT group; *n =* 50, *M* age = 37.62, SD = 10.67 years; 30 females), and the remaining forty-five employees served as a no-training control group (NTC group; *n* = 45; *M* age = 40.51, SD = 11.85 years; 37 females). MBAT was delivered in person by a context-familiar, non-research affiliated trainer who was a member of the organization. During the 4-week training interval, MBAT participants were assigned 15 min of formal, out-of-class MBAT practices. This study used a non-randomized design and had the primary goal of examining the efficacy of MBAT delivery by recently trained organizational trainers (see Denkova et al., 2022 for more details).

#### Study 4

Seventy-six community leaders from a small-yet-prominent city, coming from various sectors, such as business, healthcare, education, public safety, and non-profit organizations, participated in this study. Of the seventy-six, forty-one leaders volunteered to participate in MBAT (MBAT group; *n =* 41, *M* age = 50.59, SD = 12.72 years; 31 females). A few months later, thirty-five leaders served as a no-training control group (NTC group; *n* = 35; *M* age = 51.61, SD = 9.67 years; 30 females). In this study, participants engaged in 2, 4-h MBAT sessions over 2 days delivered in person by a research- affiliated trainer. In the following 4 weeks participants were assigned daily 15-min practice and offered the opportunity to attend “office hours” in which they could meet with the trainer via teleconference session to discuss their experiences and ask questions regarding course content and materials. MBAT themes were contextualized for the community leader environment. For example, the connection theme addressed adaptive and effective leadership, explored team cohesion, and the cultivation of kindness/connection practices involving the intention of kindness to be directed towards oneself, a fellow leader in the participant’s occupational environment, and their organizational team as a whole. This study used a non-randomized design and had the primary goal of examining the efficacy of MBAT in community leaders.

#### Study 5

Using a cluster-randomized design, fifty-one educators from a co-educational school in South Florida were assigned by their work location (e.g., school campus) to receive mindfulness training (MBAT group; *n* = 30; *M* age = 48.03, SD = 8.98 years, 24 females), or to a no-training control group (NTC group; *n* = 21; *M* age = 41.95, SD = 10.68 years; 18 females). The NTC group received MBAT after the second testing session (T2). MBAT themes were contextualized by incorporating educational (i.e., classroom) terminology and cultural references, and examples relatable to those working within an educational setting. MBAT was also delivered in person by a research-affiliated trainer. Training group participants were assigned 10–15 min of daily MBAT practices to be completed outside of class sessions. In addition, training group participants were encouraged to incorporate informal practices offered each week into their daily lives.

### Procedure

Participants in all five studies completed two testing sessions (T1 and T2) separated by a 4-week interval over which the training groups received the MBAT program, and no-training control groups did not. Studies 1, 2, and 5 employed in-person testing proctored by 1 or 2 experimenters in a group setting with up to 10 participants (see Denkova et al., 2020, 2021). In studies 3 and 4, participants engaged in remote testing sessions through Inquisit Web (Millisecond Software, LLC), which is an online platform that facilitates remote data collection for research purposes. During each testing session spanning approximately ninety minutes, participants were instructed to complete a battery of tests in one sitting. Participants were also instructed to complete testing in a quiet space where they could minimize possible distractions and interruptions. Further, Inquisit locks participants’ computers from opening/accessing any other screens during the duration of testing, thus minimizing potential distractions and interruptions. All testing sessions included a variant of the SART with embedded probes indexing subjective probe-caught mind wandering and meta-awareness (Robertson et al., 1997), and a series of self-reported questionnaires related to psychological health and emotional well-being.

### Measures

#### Sustained Attention to Response Task

**Figure 1:**
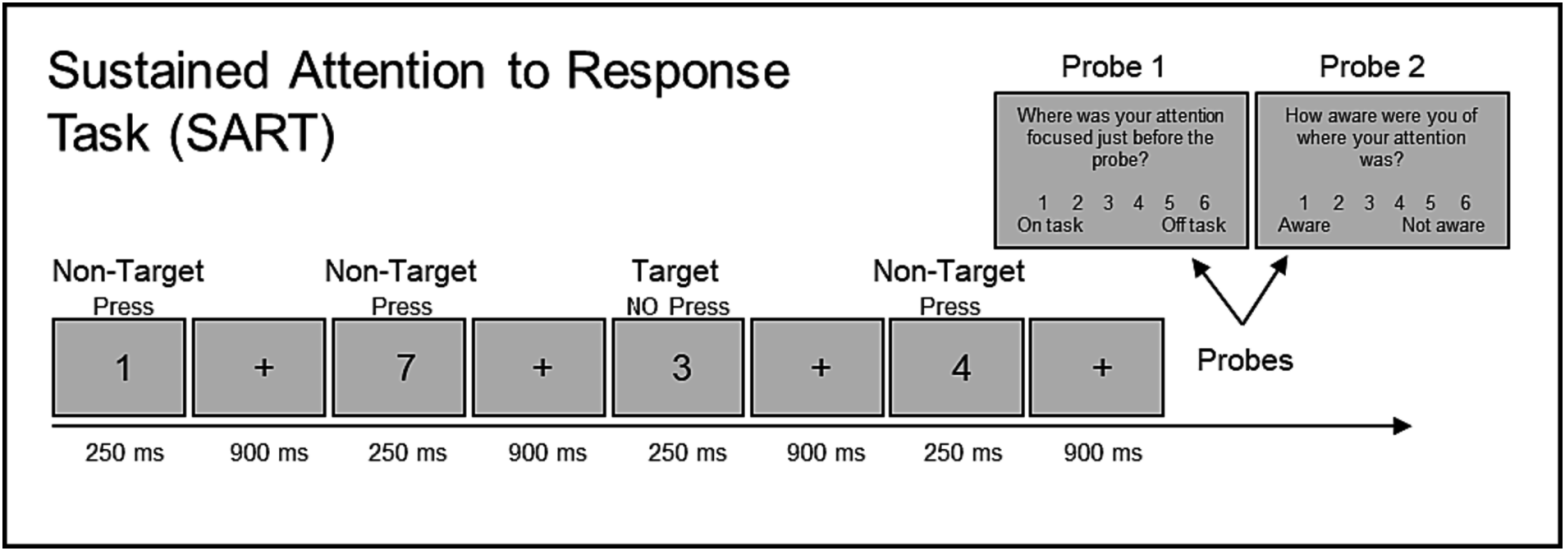
Demonstrates the design of the Sustained Attention to Response Task (SART). Single digits (0 through 9) were continuously presented on screen one at a time for 250 msec followed by an inter-trial-interval of 900 msec during which a fixation cross was presented. Participants were instructed to refrain from pressing the spacebar to the target number 3 (5% of trials) and to press the spacebar for all other non-target digits. Experience sampling probes intermittently interrupted task performance to ask participants to respond to two probe questions using a Likert-like 6-point scale.

The Sustained Attention to Response Task (SART, Robertson et al., 1997) is a go/no-go task that is typically used as a measure of sustained attention. During the SART, single digits (0 through 9) were presented for 250 ms, and each digit was followed by an inter-trial interval with a fixation cross for 900 ms (Figure 1). Participants were instructed to withhold pressing the spacebar in response to the digit 3 (target) and to press the spacebar for all other digits (non-targets) as quickly as possible without sacrificing accuracy. Responses were recorded during the digit display, as well as the inter-trial interval. Target trials occurred very infrequently on about 5% of the experimental trials.

On occasion, two probe questions were presented in succession and distributed throughout the task. Across studies, on average, there were 20.9 trials between probes.^1^ Participants were told that probe questions will occasionally ask about the focus of their attention. The first probe question (probe 1) asked, “Where was your attention focused just before the probe?” Participants were instructed to respond on a 6-point scale ranging from 1 (on task) to 6 (off task). Probe 1 is referred to as the Mind Wandering probe. The second probe question (probe 2) asked, “How aware were you of where your attention was?” Participants were instructed to respond on a 6-point scale ranging from 1 (aware) to 6 (unaware). Probe 2 is referred to as the Meta-Awareness probe. The probe questions were displayed until a response was made.

After the practice block, participants were instructed to complete two experimental blocks comprising target and nontarget trials and respond to probes presented in pseudorandom order. The specific number of trials and probes varied slightly across studies.^2^ Task metrics included subjective probe responses and objective SART outcomes. Subjective probe responses were measured by separately calculating the mean of probe ratings for each probe question. Objective SART outcomes included task accuracy indexed by *A* ′ and variability in response time (RT). *A* ′ is a nonparametric measure of sensitivity (Stanislaw and Todorov, 1999), which yields a composite of hits (correctly withholding a response to target trials) and false alarms (incorrectly withholding a response to non-target trials). Variability in RT is indexed by the intra-individual coefficient of variation (ICV), which is calculated as the standard deviation of RTs for correct nontarget trials divided by the mean RT of correct non-target trials (i.e., for each participant: standard deviation RT/mean RT).

### Statistical analysis

#### Data inclusion

Exclusion criteria for this meta-analysis were identical across all five contributing studies. While some of the prior published studies utilized an intent-to-treat approach for statistical analyses, including participants with missing data at one time point, we only included participants with complete data at both time points in the present meta-analyses because this facilitated the calculation of standardized effect sizes and aggregation of effects using meta-analysis methods. Of the 417 participants with T1 data in all five studies, 91 participants did not provide data at T2, and therefore, were not included in these analyses. An additional 22 participants were excluded from analyses due to problems with assessing their task performance because of a lack of adherence to task instructions or below chance performance (*A’* < 0.5) at T1 or T2. No other outliers were excluded from analyses. To allow for comparisons across studies, only participants in the MBAT and no-training conditions were included in analyses, and those in active comparison conditions (i.e., relaxation training, Study 1) were excluded. There was slight variability in rates of missing data across studies. Table 2 provides final reported sample sizes and descriptive statistics for dependent measures for each study.

**Table 2:** Descriptive statistics.

		Probe 1					Probe 2					A′					ICV				
		T1		T2			T1		T2			T1		T2			T1		T2		
Condition	*N*	Mean (SD)		Mean (SD)		*r*	Mean (SD)		Mean (SD)		*r*	Mean (SD)		Mean (SD)		*r*	Mean (SD)		Mean (SD)		*r*
Study 1																					
MBAT	34	1.62	(1.01)	1.51	(0.67)	0.324	1.76	(1.15)	1.67	(1.01)	0.600	0.90	(0.05)	0.90	(0.07)	0.426	0.29	(0.09)	0.29	(0.11)	0.453
NTC	42	1.60	(0.64)	1.68	(0.79)	0.738	1.61	(0.61)	1.76	(0.89)	0.617	0.89	(0.06)	0.90	(0.06)	0.473	0.29	(0.09)	0.27	(0.12)	0.640
Study 2																					
MBAT	40	1.78	(0.59)	1.73	(0.63)	0.788	1.79	(0.67)	1.68	(0.67)	0.501	0.89	(0.06)	0.90	(0.06)	0.526	0.26	(0.10)	0.25	(0.09)	0.661
NTC	41	1.63	(0.55)	1.84	(0.62)	0.536	1.61	(0.57)	1.77	(0.68)	0.654	0.86	(0.11)	0.87	(0.10)	0.577	0.30	(0.10)	0.33	(0.21)	0.455
Study 3																					
MBAT	29	1.56	(0.56)	1.56	(0.50)	0.360	1.49	(0.50)	1.52	(0.51)	0.588	0.88	(0.06)	0.92	(0.06)	0.588	0.28	(0.08)	0.21	(0.06)	0.465
NTC	18	1.76	(0.66)	2.14	(0.82)	0.760	1.63	(0.55)	1.86	(0.55)	0.602	0.87	(0.07)	0.88	(0.09)	0.709	0.26	(0.09)	0.27	(0.08)	0.606
Study 4																					
MBAT	29	1.90	(0.49)	1.77	(0.49)	0.600	1.88	(0.64)	1.69	(0.70)	0.784	0.90	(0.06)	0.93	(0.05)	0.553	0.24	(0.07)	0.25	(0.06)	0.670
NTC	26	1.84	(0.78)	2.03	(0.73)	0.620	1.85	(0.95)	2.03	(0.91)	0.774	0.90	(0.07)	0.92	(0.09)	0.715	0.24	(0.12)	0.22	(0.11)	0.895
Study 5																					
MBAT	25	1.67	(0.52)	1.61	(0.56)	0.543	1.47	(0.44)	1.50	(0.49)	0.607	0.91	(0.07)	0.92	(0.07)	0.661	0.27	(0.07)	0.25	(0.09)	0.443
NTC	20	1.98	(0.73)	2.05	(0.99)	0.593	1.88	(0.82)	2.07	(1.02)	0.499	0.88	(0.07)	0.90	(0.06)	0.098	0.30	(0.09)	0.30	(0.08)	0.234

Descriptive statistics are provided for all participants with complete data at both study time points including sample size (*N*), means, standard deviations, and bivariate correlations.

#### Meta-analytic procedures

Measures of standardized effect size were calculated using the package *metafor* in R (Viechtbauer, 2010) from summary descriptive statistics for each of the five studies (see Table 2). First, the standardized mean change (SMC) from T1 to T2 for each condition (MBAT and NTC groups) was calculated for each of the five studies. A negative SMC reflects an attenuation in task accuracy (*A’*), reduced response time variability (ICV), a decrease in self-reported mind wandering, and greater meta-awareness of one’s off-task thoughts from T1 to T2. Conversely, a positive SMC reflects improvement in task accuracy (*A’*), an increase in response time variability (ICV), an increase in mind wandering, and a decreased awareness of one’s mind wandering from T1 to T2.

SMC values for each dependent measure were aggregated across the five studies in a multivariate mixed effects meta-analysis, which calculated a conditional weighted SMC for MBAT and NTC groups. Group effects were nested within their corresponding study, and study condition (MBAT vs. NTC) was included as a moderator variable to estimate the separate effects for MBAT and NTC groups. The model employed maximum likelihood estimation, and model effects were weighted according to the inverse variance.

To obtain the meta-analytic effect of MBAT on SART outcomes, a standardized effect size was calculated that reflects the difference (Δ) between MBAT and NTC groups’ SMC from T1 to T2. An overall weighted ΔSMC was estimated across studies with a random effects model estimated using restricted maximum likelihood with weighting based on the inverse variance. The 95% prediction interval (95% PI) around the SMC and ΔSMC were also calculated, which reflects the range of expected effects observed from future studies. Finally, to measure the proportion of variance in the model explained by heterogeneity among the included studies, *I^2^* and Cochran’s *Q* were calculated (Higgins et al., 2003). In addition, funnel plots were reviewed to evaluate the symmetry of effects, as well as the statistical power of each study to detect the meta-analytic effect.

## Results

The meta-analysis of the five included studies (*N =* 304) identified a significant difference (ΔSMC) between MBAT and NTC groups over time (i.e., from T1 to T2) for mind wandering, meta-awareness, and ICV, but did not reveal a significant difference for *A′*. Descriptive statistics for each study may be found in Table 2. The results of the meta-analysis are described below.

### Mind wandering

**Figure 2:**
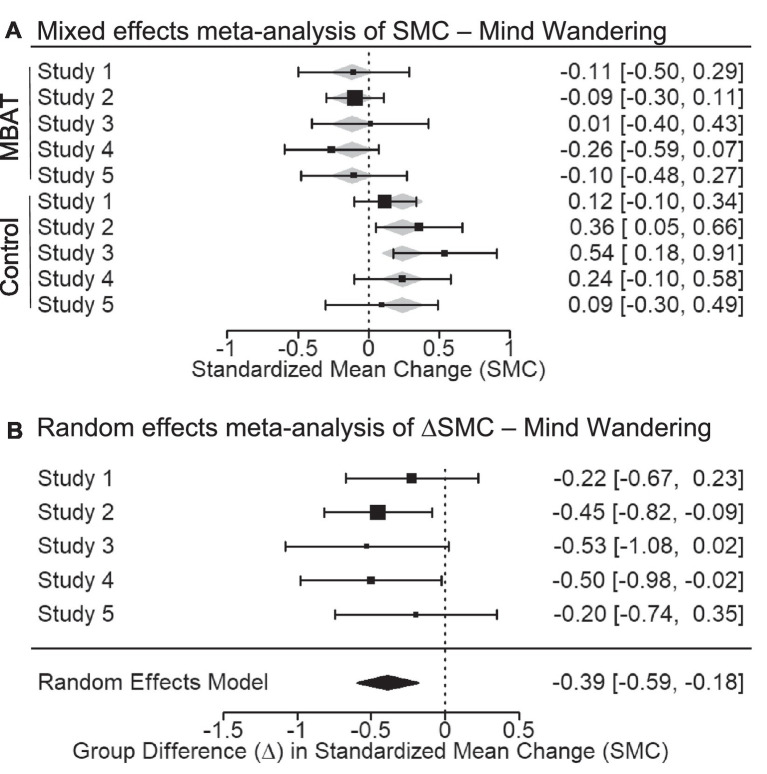
**(A)** Depicts the multivariate mixed effects meta-analysis of standardized mean change (SMC) from T1 to T2 in probe-caught mind wandering scores for MBAT and control conditions. Negative SMCs indicate that individuals mind wander less frequently from T1 to T2 with meta-analytic effect sizes and 95% confidence intervals shown as black boxes and error bars. **(B)** Depicts the difference in standardized mean change between MBAT and control groups (∆SMC) in probe-caught mind wandering scores. A negative ∆SMC indicates greater decreases in mind wandering scores from T1 to T2 in MBAT groups relative to control groups. Based on the random effects model, the overall meta-analytic effect size and 95% confidence interval is provided below the individual study estimates.

In a fixed effects meta-analysis of Probe 1, which measured the average self-reported mind wandering (probes rated “on-task” to “off-task”), the conditional SMC for MBAT groups did not significantly differ from zero (SMC = −0.113, *p* = 0.108, 95% CI [−0.251, 0.025], 95% PI [0.097, 0.389]). The 95% confidence interval around this effect overlapped with a small effect size (−0.251 to 0.025). In contrast, the SMC for NTC groups was significantly different from zero (SMC = 0.243, *p* < 0.001, 95% CI [0.105, 0.381], 95% PI [−0.251, 0.250]), indicating a small increase in subjective mind wandering over time. Figure 2A illustrates the SMCs for each study, as well as the mixed effects weighted estimates for MBAT and NTC groups.

Random effects meta-analysis of the difference between MBAT and NTC groups in standardized mean change (ΔSMC) was significant (ΔSMC = −0.387, *p* < 0.001, 95% CI [−0.594, −0.181], 95% PI [−0.594, 0.181]). ^3^ Together, these results indicate that MBAT was associated with a − 0.387 ΔSMC between MBAT and NTC groups, which suggests that, over time, while participants in the NTC group increased in their mind wandering, participants in the MBAT group were protected against such increases. These patterns of change-over-time in mind wandering were small to medium in size.

**Figure 3:**
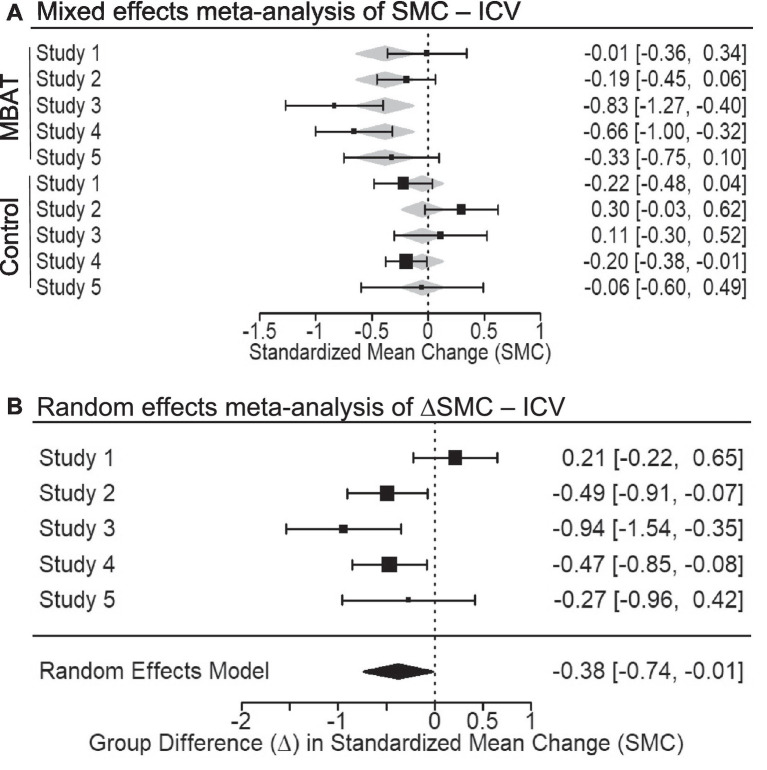
Funnel plots of the group difference in standard mean change (∆SMC) for **(A)** mind wandering, **(B)** meta-awareness, **(C)** ICV, and **(D)** *A′*. ∆SMCs for each study are depicted as white dots and are plotted by the standard error from each respective study. The color gradient represents the overall statistical power of the meta-analytic effect from red (low power) to green (high power). The vertical line that bisects the triangle depicts the meta-analytic effect size for each of the outcomes. Estimates in the shaded region fall within the 5 to 1% significance level from zero.

Figure 2B depicts the ΔSMCs of each study, as well as the random effects weighted estimates for Mind Wandering scores among MBAT and NTC groups. Evaluation of *I^2^* and the *Q* statistic suggested that studies were largely homogenous in their magnitude of effects (*I^2^* < 1%, *Q* = 1.565, *p* = 0.815). A funnel plot illustrating the ΔSMCs across all studies for mind wandering is depicted in Figure 3A. Based on this plot, studies appear generally symmetrically distributed around the meta-analytic effect size (ΔSMC = −0.387). The funnel plot also depicts the statistical power of each study to detect the meta-analytic effect. The median power of studies was 35.6%, suggesting that studies were largely underpowered to detect an effect size of this magnitude.

### Meta-awareness

**Figure 4:**
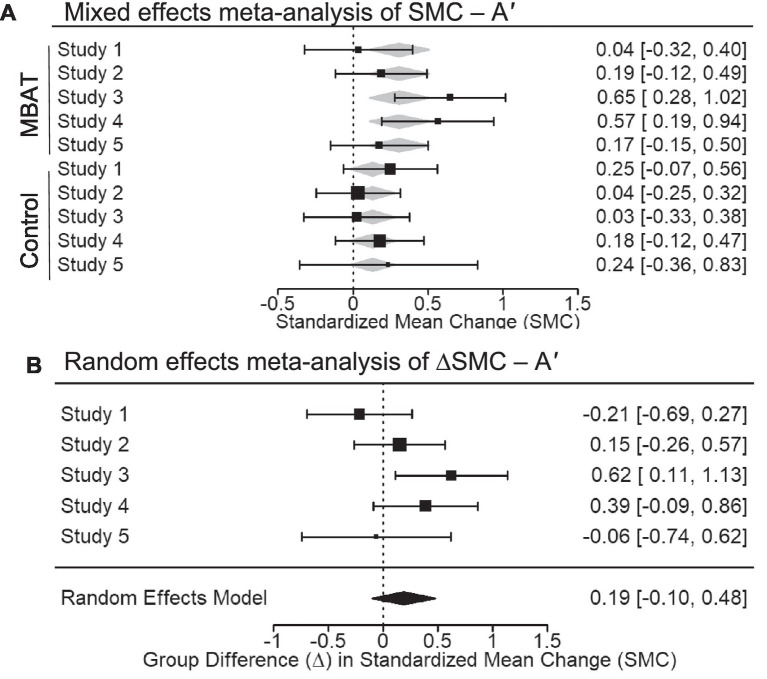
**(A)** Depicts the multivariate mixed effects meta-analysis of standardized mean change (SMC) from T1 to T2 in probe-caught meta-awareness scores for MBAT and control conditions. Negative SMCs indicate that individuals are more aware from T1 to T2 with meta-analytic effect sizes and 95% confidence intervals shown as black boxes and error bars. **(B)** Depicts the difference in standardized mean change between MBAT and control groups (∆SMC) in meta-awareness scores. A negative ∆SMC indicates greater increases in awareness from T1 to T2 in MBAT groups relative to control groups. Based on the random effects model, the overall meta-analytic effect size and 95% confidence interval is provided below the individual study estimates.

In a fixed effects meta-analysis of Probe 2, which measured awareness of subjective mind wandering (probes rated “completely aware” to “completely unaware”), the conditional SMC for MBAT did not significantly differ from zero (SMC = −0.118, *p* = 0.096, 95% CI [−0.256, 0.021], 95% PI [−0.256, 0.021]). The 95% confidence interval around this effect overlapped with small effect sizes (−0.256 to 0.021). In contrast, the SMC for NTC groups was significantly different from zero (SMC = 0.249, *p* < 0.001, 95% CI [0.112, 0.386], 95% PI [0.112 to 0.386]), indicating a small decrease in awareness over time. Figure 4A illustrates the SMCs for each study, as well as the mixed effects weighted estimates for MBAT and NTC groups.

Random effects meta-analysis of the difference between MBAT and NTC groups in standardized mean change (ΔSMC) was significant (ΔSMC = −0.374, *p* < 0.001, 95% CI [−0.571, −0.177], 95% PI [−0.571 to −0.177]).^4^ These results indicate that MBAT and NTC groups had a small difference in their standardized mean change over time (ΔSMC = −0.374), suggesting that participants in the MBAT group had significantly different patterns of change over time compared to the NTC group. While the NTC group declined in their meta-awareness over time, participants in the MBAT group did not demonstrate significant reductions. These patterns of change-over-time in meta-awareness were small to medium in size.

Figure 4B depicts the ΔSMCs of each study, as well as the random effects weighted estimates for Meta-Awareness scores among MBAT and NTC groups. Evaluation of *I^2^* and the *Q* statistic suggested that studies were largely homogenous in their magnitude of effects (*I^2^* < 1%, *Q* = 1.050, *p* = 0.902). A funnel plot illustrating the ΔSMCs for meta-awareness across all studies is depicted in Figure 3B. Based on this plot, studies appear roughly symmetrically distributed around the meta-analytic effect size (ΔSMC = −0.374). The funnel plot also depicts the statistical power of each study to detect the meta-analytic effect. The median power of studies was 43.7%, suggesting that studies were generally underpowered to detect an effect size of this magnitude.

### Intra-individual coefficient of response time variability (ICV)

**Figure 5:**
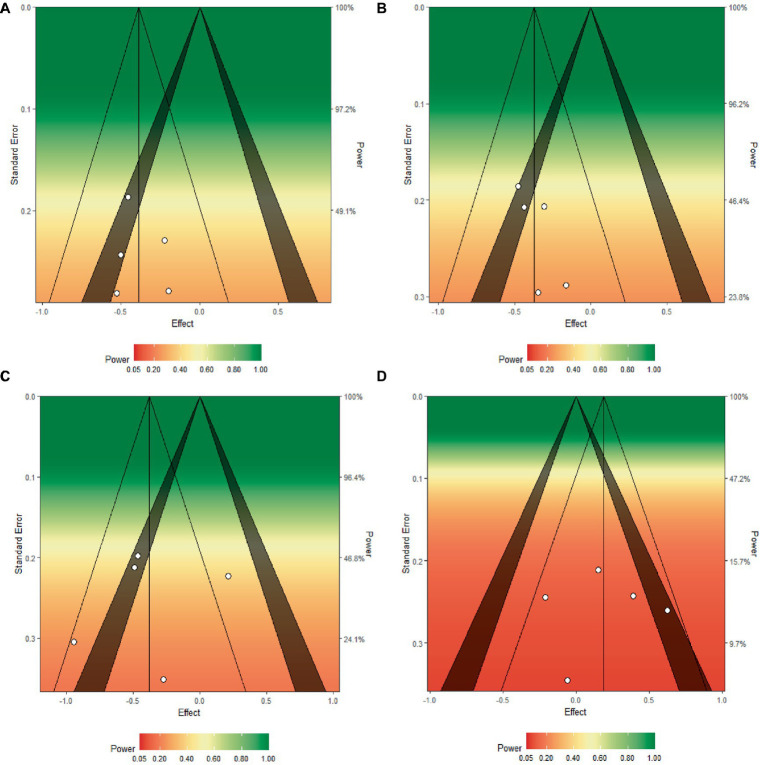
**(A)** Depicts the multivariate mixed effects meta-analysis of standardized mean change (SMC) from T1 to T2 in response time variability (ICV) for MBAT and control conditions. Negative SMCs indicate a decrease in ICV from T1 to T2 with meta-analytic effect sizes and 95% confidence intervals shown as black boxes and error bars. **(B)** Depicts the difference in standardized mean change between MBAT and control groups (∆SMC) in ICV. A negative ∆SMC indicates greater reduction in ICV from T1 to T2 in MBAT groups relative to control groups. Based on the random effects model, the overall meta-analytic effect size and 95% confidence interval is provided below the individual study estimates.

In a fixed effects meta-analysis of ICV, which measured response time variability, the conditional SMC for MBAT significantly differed from zero (SMC = −0.386, *p* < 0.01, 95% CI [−0.642, −0.130], 95% PI [−0.900 to 0.128]). The 95% confidence interval around this effect overlapped with small to medium effect sizes (−0.642 to −0.130), indicating an overall decrease in ICV over time. The SMC for NTC groups was not significantly different from zero (SMC = −0.051, *p* = 0.587, 95% CI [−0.236, 0.133], 95% PI [−0.375 to 0.272]). Figure 5A illustrates the SMCs for each study, as well as the mixed effects weighted estimates for MBAT and NTC groups.

Random effects meta-analysis of the difference between MBAT and NTC groups in the standardized mean change for ICV was significant (ΔSMC = −0.376, *p* = 0.043, 95% CI [−0.741, −0.011], 95% PI [−1.117 to 0.365]).^5^ Together, these results indicate that MBAT and NTC groups had a small difference in their standardized mean change over time (ΔSMC = −0.376), suggesting that participants in the MBAT group had significantly different patterns of change compared to the NTC group. While participants in the MBAT group had reduced ICV, those in the NTC group increased over time. These patterns of change-over-time in ICV were small to medium in size.

Figure 5B depicts the ΔSMCs of each study, as well as the random effects weighted estimates for ICV scores among MBAT and NTC groups. Evaluation of the *I^2^* and the *Q* statistic suggested that, for ICV, studies were moderately heterogeneous in their magnitude of effects (*I^2^* = 64.36%, *Q* = 10.981, *p* = 0.027). A funnel plot of results (Figure 3C) illustrates that included studies are slightly symmetrically distributed around the meta-analytic effect size (ΔSMC = −0.376). The funnel plot also depicts the statistical power of each study to detect the meta-analytic effect. The median power of studies was 39.1%, indicating that studies were largely underpowered to detect an effect size of this magnitude.

### Accuracy (*A′*)

**Figure 6:**
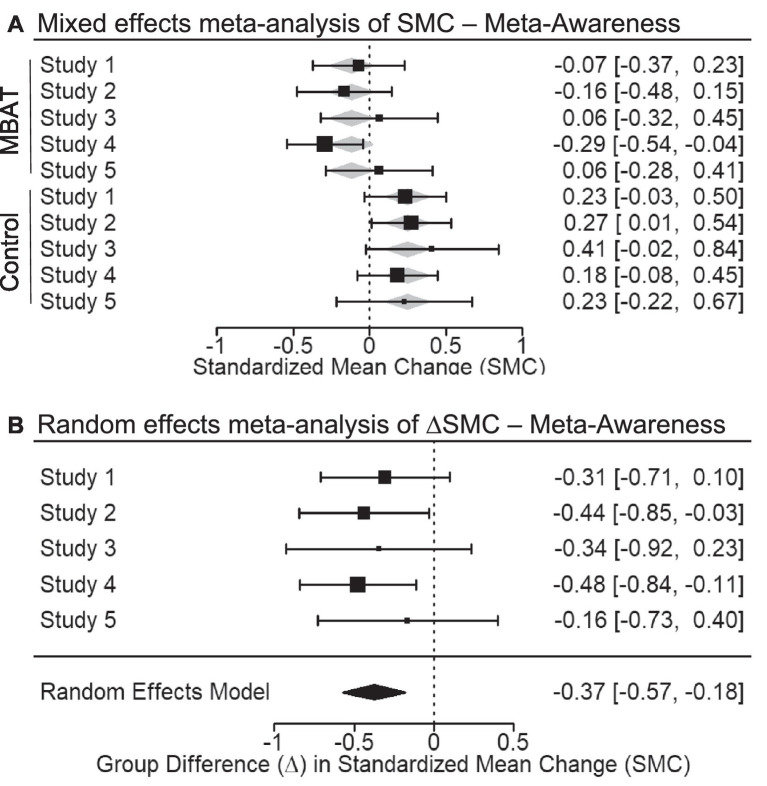
**(A)** Depicts the multivariate mixed effects meta-analysis of standardized mean change (SMC) from T1 to T2 in *A′* for MBAT and control conditions. Positive SMCs indicate an increase in *A′* from T1 to T2 with meta-analytic effect sizes and 95% confidence intervals shown as black boxes and error bars. **(B)** Depicts the difference in standardized mean change between MBAT and control groups (∆SMC) in *A′*. A positive ∆SMC indicates greater increase in *A′* from T1 to T2 in MBAT groups relative to control groups. Based on the random effects model, the overall meta-analytic effect size and 95% confidence interval is provided below the individual study estimates.

In a fixed effects meta-analysis of *A′*, measuring task accuracy, the conditional SMC for MBAT significantly differed from zero (SMC = 0.309, *p* = 0.003 95% CI [0.108, 0.510], 95% PI [−0.042 to 0.660]). The 95% confidence interval around this effect overlapped with small to medium effect sizes (0.108 to 0.510), indicating a small increase in accuracy over time. In contrast, the SMC for NTC groups did not significantly differ from zero (SMC = 0.131 *p* = 0.087, 95% CI [−0.019, 0.282], 95% PI [−0.027 to 0.289]). Figure 6A illustrates the SMCs for each study, as well as the mixed effects weighted estimates for MBAT and NTC groups.

Random effects meta-analysis of the difference between MBAT and NTC groups in the standardized mean change for *A′* was non-significant (ΔSMC = 0.189, *p* = 0.202, 95% CI [−0.101, 0.479], 95% PI [−0.315 to 0.693]).^6^ Together, these results suggest that, across studies, MBAT and NTC groups did not differ in their standardized mean change over time (ΔSMC = 0.189).

Figure 6B depicts the ΔSMCs of each study, as well as the random effects weighted estimates for *A′* scores among MBAT and NTC groups. Evaluation of the *I^2^* and the *Q* statistic suggested that, for *A′*, studies were somewhat heterogeneous in their magnitude of effects (*I^2^* = 40.71%, *Q* = 6.706, *p* = 0.152). A funnel plot of results suggests that included studies are slightly symmetrically distributed around the meta-analytic effect size (ΔSMC = 0.189), and the median power of studies was 12.1%, indicating that studies were largely underpowered to detect an effect size of this magnitude (see Figure 3D).

## Discussion

We investigated the effects of a short-form MT program on self-reported mind wandering and meta-awareness, as well as objective performance during performance of a sustained attention task. An internal meta-analysis was conducted across five studies. Small-yet-significant differences between groups in standardized mean change (ΔSMC) from pre- to post-training were found for mind wandering and meta-awareness. Specifically, while the no-training groups showed increases in mind wandering and reductions in meta-awareness over time (i.e., T1 to T2), those who received MBAT did not change over time in mind wandering or meta-awareness. These findings suggest that compared to the no-training groups, MBAT groups demonstrated functional stability which can be interpreted as a protection against increase in self-reported mind wandering and decrease in meta-awareness (see Jha et al., 2017).

In addition to the protective effects on mind wandering, our findings revealed greater attentional stability, as reflected in less variable response time variability over time in the MBAT groups. Indeed, while the no-training control participants did not significantly change in their response time variability during the SART from T1 to T2, the MBAT participants had reduced response time variability from T1 to T2 (SMC = −0.386). This is consistent with findings from several studies of MT that have reported reductions in response time variability during tasks of sustained attention following training (e.g., van den Hurk et al., 2010; Mrazek et al., 2012; Zanesco et al., 2013; Morrison et al., 2014). While the effects for ICV were small, the confidence intervals around this effect were large.

While we observed significant group differences in change-over-time in mind wandering, meta-awareness, and ICV, we did not observe such effects in SART *A’* scores. Significant changes in *A′* from T1 to T2 for the MBAT condition (SMC = 0.309) were observed. However, the magnitude of this change did not significantly differ from the change-over-time found in the NTC group.

As is often the case with early-stage intervention studies, sample sizes were small. Analyses of statistical power indicated that overall, the five studies analyzed herein were underpowered relative to reported effect sizes. The median power of studies contributing to *A′* was 12.1%, while the median power of studies contributing to mind wandering, meta-awareness, and ICV was 35.6, 43.7, and 39.1%, respectively. Our meta-analytic approach aims to address the small sample size of individual studies by aggregating effects across studies and increasing our statistical power to detect differences between MBAT and control groups.

By protecting against increases in mind wandering, MBAT may be a useful tool for reducing errors in time-pressured, applied contexts. In addition, as suggested in recent studies, these changes may mediate improvements in other psychological outcomes. A growing literature suggests that mind wandering may be implicated in fluctuating affective states (Andrews-Hanna et al., 2013; Mason et al., 2013), and mind wandering has gained utility as a marker for depressive thinking (Smallwood et al., 2007), rumination (Marchetti et al., 2016), worsened mood (Song and Wang, 2012), and symptoms of stress (Seli et al., 2019), which have all been shown to predict the onset of psychological disorders. As such, protecting against increases in mind wandering may also protect against psychological health challenges.

It is important to mention that the present study defined and operationalized mind wandering in the context of an ongoing task when its occurrence hinders task performance, as revealed in several studies and recent meta-analyses on this topic (see Randall et al., 2014; Bonifacci et al., 2023). In contrast, other studies have defined and operationalized mind wandering in a task-free context resulting in phenomena such as daydreaming, creative thinking, and other aspects of spontaneous thought (e.g., Christoff et al., 2016), which may have positive impacts (Gericke et al., 2022). While there is active research examining the boundary conditions under which mind wandering and affiliated forms of spontaneous thought may have deleterious vs. salutary effects (see Mooneyham and Schooler, 2013; Zeitlen et al., 2022), there is far less debate regarding the costs of mind wandering when it competes with task performance in real world organizational settings (see Thomson et al., 2014).

While study results favor the view that continued investigation of MBAT via larger-scale designs is warranted, there are a number of limitations that should be considered. First, three of the five studies included herein used non-randomized designs. While the studies were aimed at examining MBAT’s early-stage ‘proof-of-concept’ feasible delivery and efficacy, it will be critical for future studies to randomly assign participants. In addition, they should make use of active control interventions, such as alternate forms of training already being implemented in the participant setting. Indeed, in many applied contexts, professionals are provided workplace interventions aimed at bolstering their wellness and work performance. Thus, it is critical that future research directly compares the effects of MBAT to such extant, active control interventions via random assignment.

Second, while we inquired whether participants had prior experience with MT, their prior experience was not accounted for in the study analyses. Given that prior meditation experience has previously been found to affect the frequency with which one experiences mind wandering episodes, this variable may reflect a potential confound (Brandmeyer and Delorme, 2018), and should be controlled for in future investigations of short-form MT. Similarly, although we attempted to assess out-of-class mindfulness practice in several of our studies, we did not investigate individual differences in mindfulness practice (see Jha et al., 2010) in the present meta-analysis. Finally, some researchers have suggested the need for caution in the use of internal meta-analyses. Indeed, while internal meta-analyses provide a powerful method to increase statistical power by aggregating results across a line of related studies, they also provide an opportunity for analytic flexibility that can result in an increased probability of detecting false positive outcomes (Vosgerau et al., 2019). We attempted to mitigate this concern by including all our relevant, available studies of MBAT in civilian, applied settings regardless of whether those studies demonstrated significant benefits in the mindfulness intervention group, including data from several unpublished studies. The aim of our internal meta-analysis was to aggregate extant studies of MBAT in these civilian applied and organizational settings in order to evaluate the overall effects of the program and motivate further research in this domain. We acknowledge that some of our outcomes may reflect false positives, and the true effect size associated with the intervention may be smaller in magnitude than observed in our meta-analysis.

In sum, the current results suggest that MBAT may hold promise as a cognitive training tool. It may protect against increases in mind wandering, while increasing attentional stability in applied and organizational settings and should be investigated further. Going forward, studies of MBAT should ensure random assignment, formally consider participants’ previous mindfulness experience, recruit larger samples, and assign well-matched active control groups. Nonetheless, the present study highlights the potential value of early-stage research with small convenience samples to spur stakeholder engagement and collaboration prior to conducting larger-scale studies. Applied research is disadvantaged by the all too common “file drawer” phenomenon of withholding reporting of studies that fail to meet the gold standard because they entail convenience samples and non-random assignment to group. Reporting early-stage research while fully acknowledging design limitations, helps to advance intervention-based applied research and ultimately supports interventions to be better positioned to achieve the “highest level of potency” (Onken et al., 2014) and avoid the fate of the implementation cliff.

## Data availability statement

The original contributions presented in the study are included in the article/supplementary material, further inquiries can be directed to the corresponding author.

## Ethics statement

The studies involving humans were approved by Institutional Review Boards at the University of Miami. The studies were conducted in accordance with the local legislation and institutional requirements. The participants provided their written informed consent to participate in this study.

## Author contributions

APJ conceived of and designed the experiments. APJ and SLR conceived of and developed the MBAT mindfulness intervention. SLR supported implementation and delivery of the MBAT mindfulness intervention. APJ, APZ, ED, and JB oversaw all aspects of data collection. MMP, APZ, APJ, and ED contributed to different stages of data analyses. All authors contributed to the article and approved the submitted version.

## Footnotes

^1^In studies 1, 2, and 5, probes were separated by 19.5 trials on average, *SD* = 9.72, range = 4–37. In studies 3 and 4, probes were separated by 23 trials on average, *SD* = 14.46, range = 7–76.

^2^Experimental blocks comprised a total of either 519 (Studies 1, 2 and 5) or 635 (Studies 3 and 4) non-target trials, either 27 (Studies 1, 2, and 5) or 32 (Studies 3 and 4) target trials, and either 28 (Studies 1, 2, and 5) or 32 (Studies 3 and 4) sets of probes. The numbers of non-target and target trials and sets of probes are slightly different due to the switch in software platform from E-Prime 3.0 (Psychology Software Tools, Pittsburgh, PA) in Studies 1, 2, and 5 to Inquisit (Millisecond Software, LLC) in Studies 3 and 4.

^3^The difference (∆) in unstandardized mean change in mind wandering was also significant (∆MC = -0.266, *p* < 0.001).

^4^The difference (∆) in unstandardized mean change in meta-awareness was also significant (∆MC = −0.264, *p* < 0.001).

^5^The difference (∆) in raw mean change in ICV (response time variability) was not significant (∆MC = −0.029, *p* = 0.085).

^6^The difference (∆) in raw mean change in *A’* (task accuracy) was not significant (∆MC = 0.0103, *p* = 0.248).
